# Laryngeal Tuberculosis Mimicking Laryngeal Carcinoma: A Case Report

**DOI:** 10.7759/cureus.87828

**Published:** 2025-07-13

**Authors:** Zineb Yammouri, Smail Chaouche, Ghita Alami, Fadoua Harmouch, Badreddine Alami, Y. Lamrani, Nawal Hammas, Meryem Boubbou, Mustapha Maaroufi

**Affiliations:** 1 Radiology, University Hospital Center Hassan II, Fez, MAR; 2 Radiology, University Hospital Center Hassan II, Sidi Mohamed Ben Abdellah University, Fez, MAR; 3 Pathology, Faculty of Medicine and Pharmacy, University Hospital Center Hassan II, Sidi Mohamed Ben Abdellah University, Fez, MAR; 4 Pathology, University Hospital Center Hassan II, Fez, MAR; 5 Radiology, Mother and Child and Interventional Imaging, University Hospital Center Hassan II, Fez, MAR

**Keywords:** caseating granuloma, contrast-enhanced ct, laryngeal carcinoma, laryngeal tuberculosis, mycobacterium tuberculosis

## Abstract

Laryngeal tuberculosis (LTB) is a rare form of extrapulmonary tuberculosis that can clinically and radiologically resemble laryngeal carcinoma, leading to potential misdiagnosis. We report the case of a 53-year-old man with a history of chronic smoking, no known tuberculosis or BCG vaccination, who presented with progressive dyspnea, dysphonia, and significant weight loss. Laryngoscopy revealed ulcerative lesions involving the anterior commissure, right ventricular strip, arytenoid fold, and epiglottis. CT imaging suggested malignancy, but a biopsy confirmed LTB with pulmonary involvement. The patient responded favorably to anti-tuberculous therapy, with near-complete resolution after two months. This case highlights the diagnostic challenge of differentiating LTB from malignancy. Awareness of this rare presentation is essential, especially in tuberculosis (TB)-endemic regions, to avoid unnecessary surgical intervention and ensure prompt medical treatment.

## Introduction

Laryngeal tuberculosis (LTB) is a rare form of extrapulmonary tuberculosis (TB), accounting for less than 1% of all TB cases [1]. Despite the global decline in TB incidence, LTB remains encountered in high-prevalence regions - particularly in Asia, Africa, and South America - or among immunocompromised individuals [1].

The larynx may be affected via direct mucosal infection, hematogenous dissemination, or secondary extension from pulmonary lesions. Clinically and radiologically, LTB often mimics laryngeal carcinoma, with shared features such as hoarseness, odynophagia, weight loss, and ulcerative or mass-like lesions on laryngoscopy [2,3]. Imaging typically reveals focal or diffuse thickening with heterogeneous enhancement, making differentiation from malignancy difficult [3].

Although laryngeal carcinoma is more frequent in older patients with risk factors such as smoking and alcohol use, TB should be considered in the differential diagnosis of laryngeal masses, particularly in endemic regions - even in the absence of systemic symptoms or confirmed pulmonary disease [4]. Histopathological examination remains essential for diagnosis, typically showing caseating granulomas with epithelioid histiocytes and Langhans-type giant cells [5].

In this report, we present a case of LTB mimicking a malignant lesion, highlighting the importance of maintaining a high index of suspicion for infectious causes in the differential diagnosis and the critical role of histopathological confirmation in guiding appropriate medical therapy.

## Case presentation

A 53-year-old man with a history of chronic smoking weaned in 1990, and no known history of pulmonary TB or Bacillus Calmette-Guérin (BCG) vaccination, presented with progressive dyspnea for three months, dysphonia for one month, and significant weight loss. He reported no fever, hemoptysis, or night sweats. Clinical examination was otherwise unremarkable.

Laryngoscopic evaluation revealed irregular ulcerative lesions involving the anterior commissure, the right ventricular strip, the right arytenoid fold, and the epiglottis. These findings raised strong suspicion of a malignant process.

**Figure 1:**
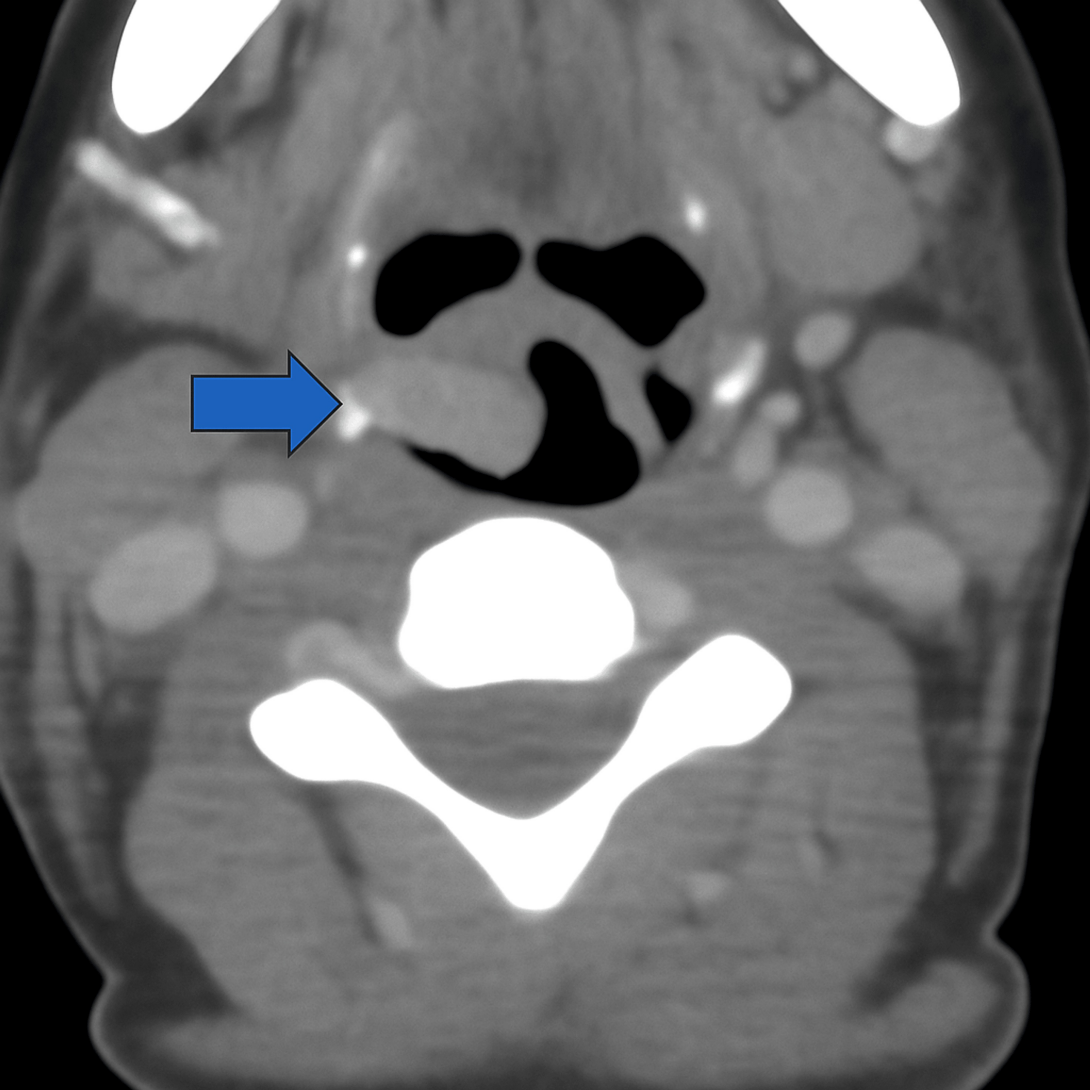
Axial CECT neck – right arytenoid fold thickening Axial contrast-enhanced CT image of the neck showing thickening of the right arytenoid fold, without associated cervical lymphadenopathy (blue arrow). CECT: Computed tomography with contrast

**Figure 2:**
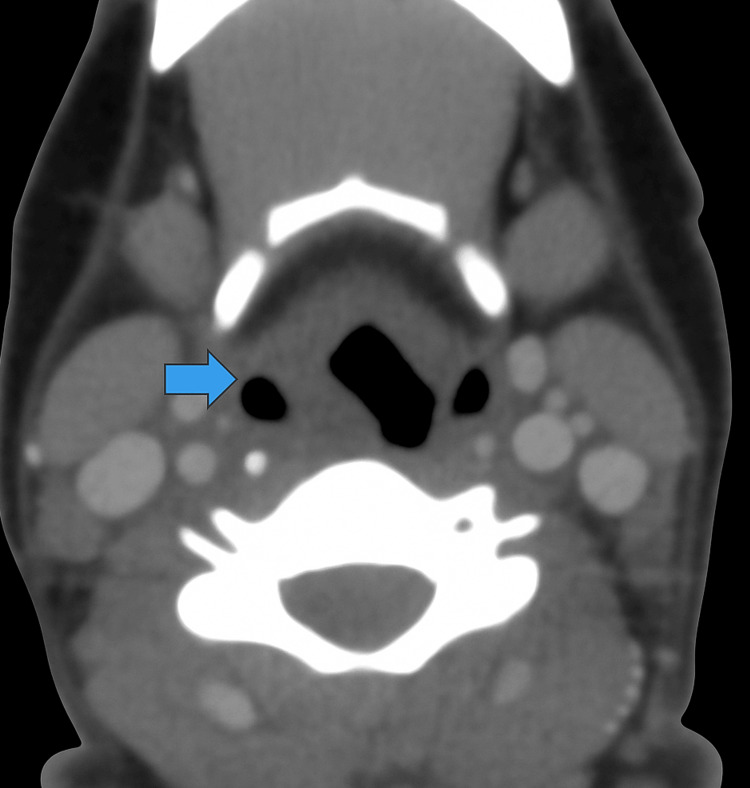
Axial CECT neck – right piriform sinus involvement Axial contrast-enhanced CT demonstrating soft tissue thickening and partial obliteration of the right piriform sinus (blue arrow). CECT: Computed tomography with contrast

**Figure 3:**
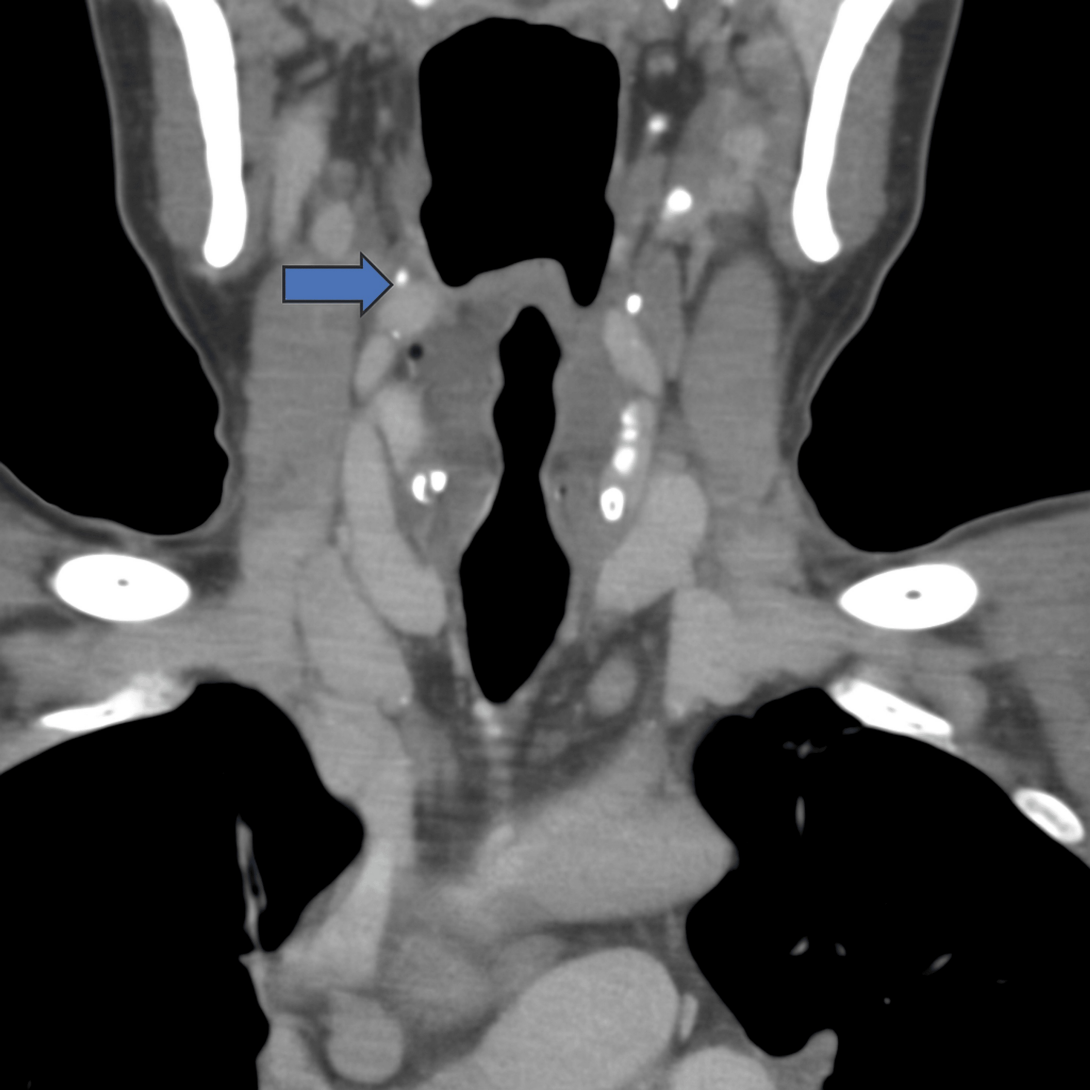
Coronal CECT neck – paraglottic space infiltration Coronal CT image of the neck showing asymmetry of the laryngeal structures with effacement and infiltration of the right paraglottic fat plane (blue arrow). CECT: Computed tomography with contrast

**Figure 4:**
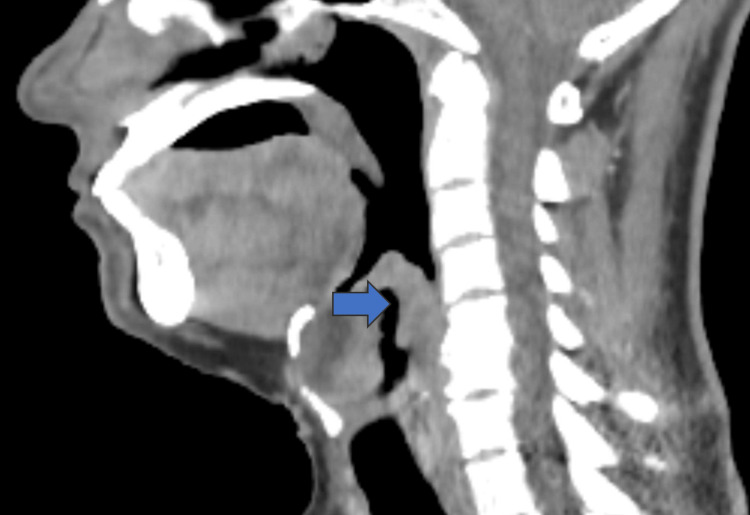
Sagittal CECT neck – supraglottic extension Sagittal contrast-enhanced CT showing cranio-caudal thickening of the supraglottic mucosa, involving the epiglottis and aryepiglottic folds, suggestive of an infiltrative lesion (blue arrow). CECT: Computed tomography with contrast

**Figure 5:**
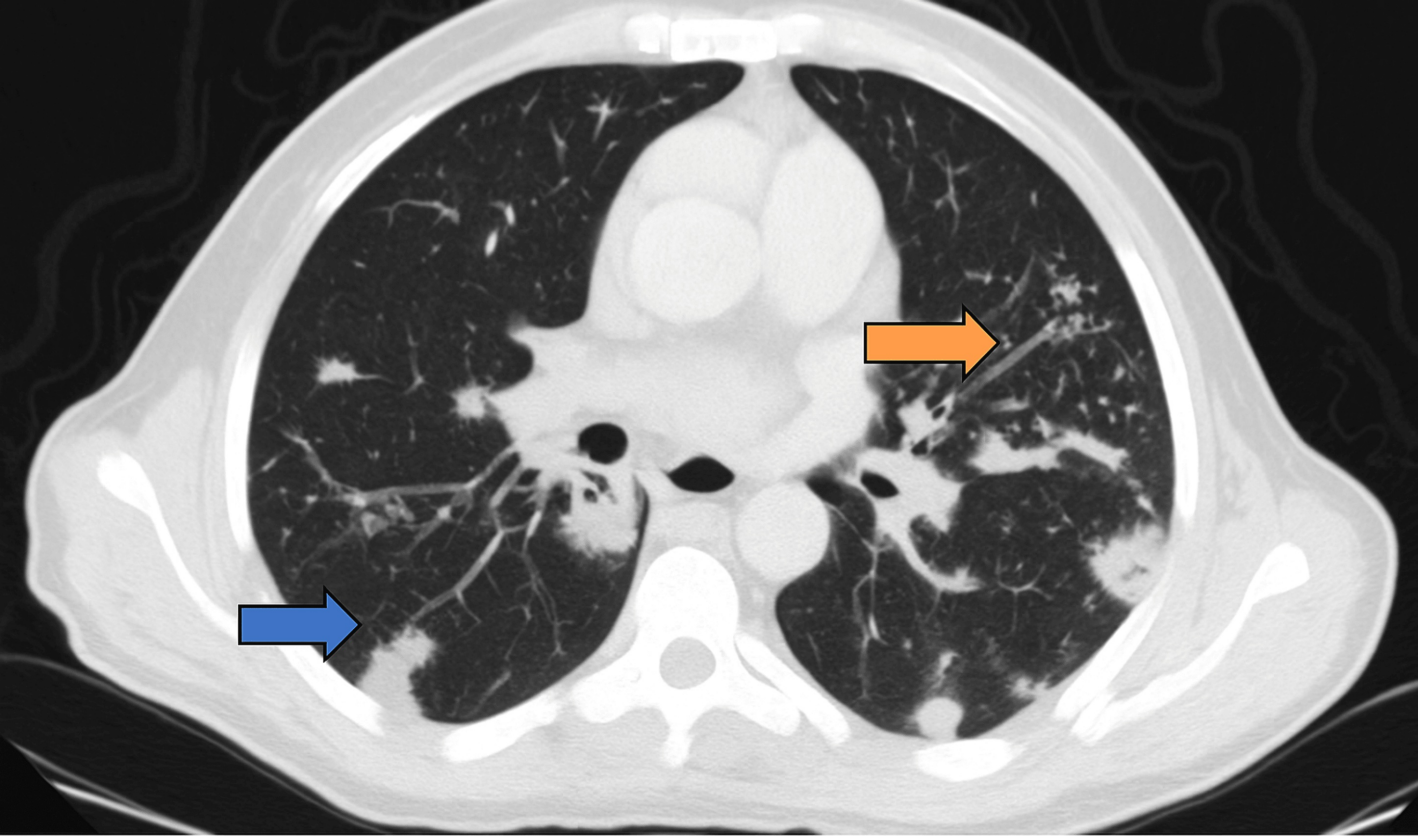
Axial chest CT – bilateral pulmonary nodules Axial chest CT (lung window) showing multiple bilateral pulmonary nodules, including cavitary lesions with spiculated margins (blue arrow), as well as branching micronodules exhibiting a tree-in-bud pattern (orange arrow), suggestive of endobronchial spread.

**Figure 6:**
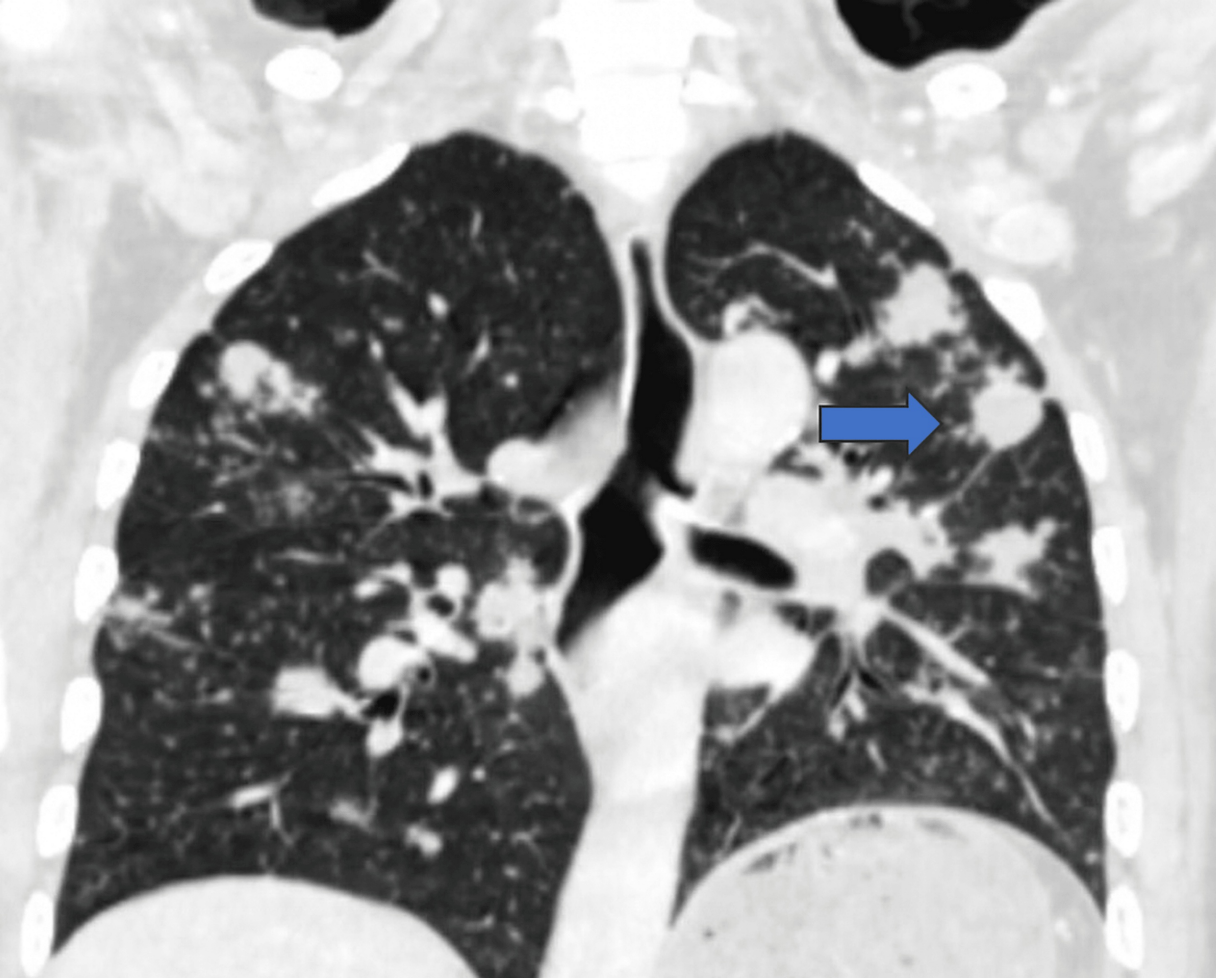
Coronal chest CT – distribution of pulmonary lesions Coronal chest CT (lung window) showing bilateral pulmonary nodules predominantly in the upper lobes, with irregular and spiculated margins (blue arrow).

A contrast-enhanced CT scan of the neck demonstrated diffuse thickening of the laryngeal structures with heterogeneous enhancement. No cervical lymphadenopathy was identified (Figures 1-4). In addition, pulmonary nodules were noted on chest imaging, which further increased the suspicion of malignancy (Figures 5-6).

**Figure 7:**
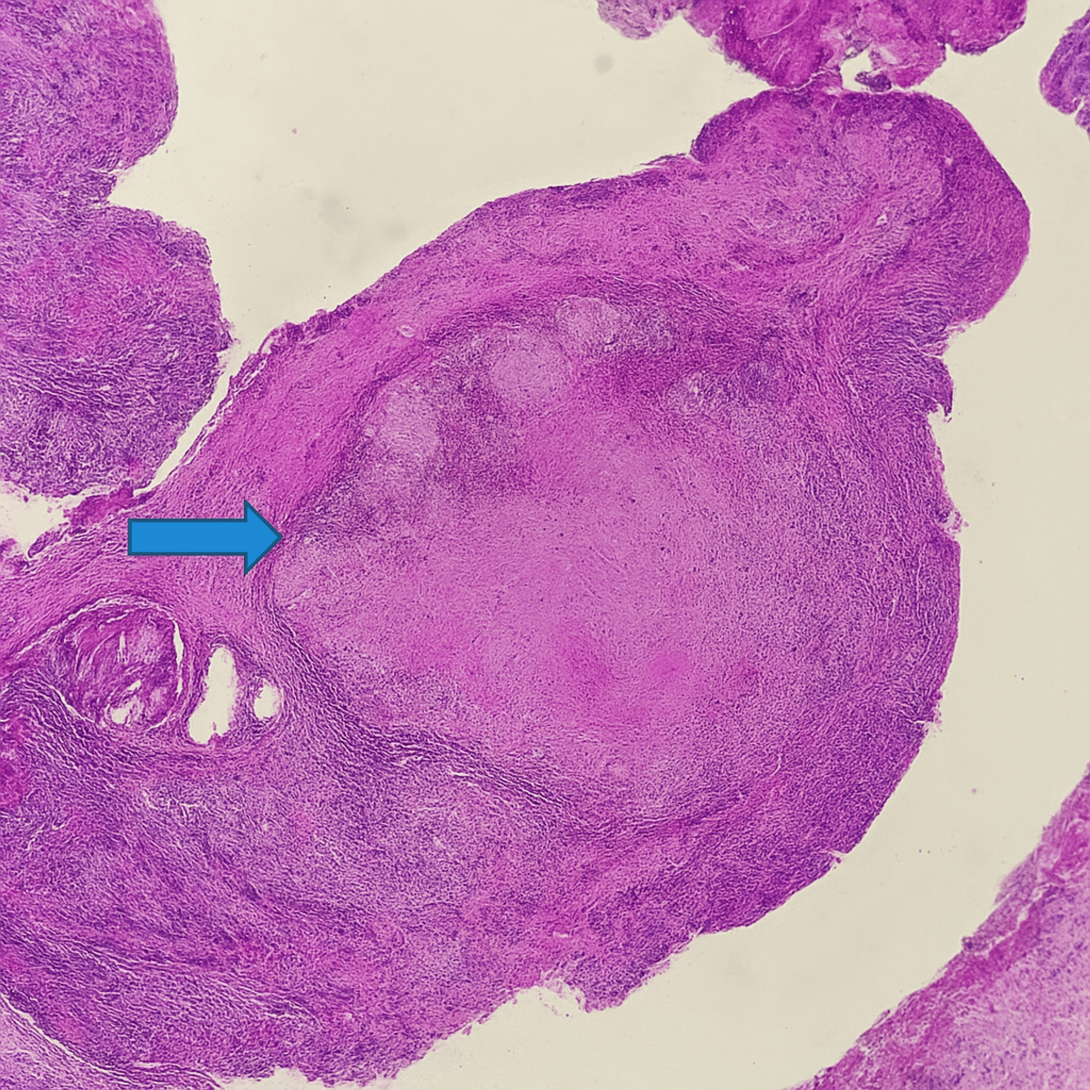
H&E stain, x40 Histopathological section showing a well-formed epithelioid granuloma with central caseous necrosis (blue arrow), characteristic of laryngeal tuberculosis. The preserved mucosal architecture helps distinguish it from invasive carcinoma.

**Figure 8:**
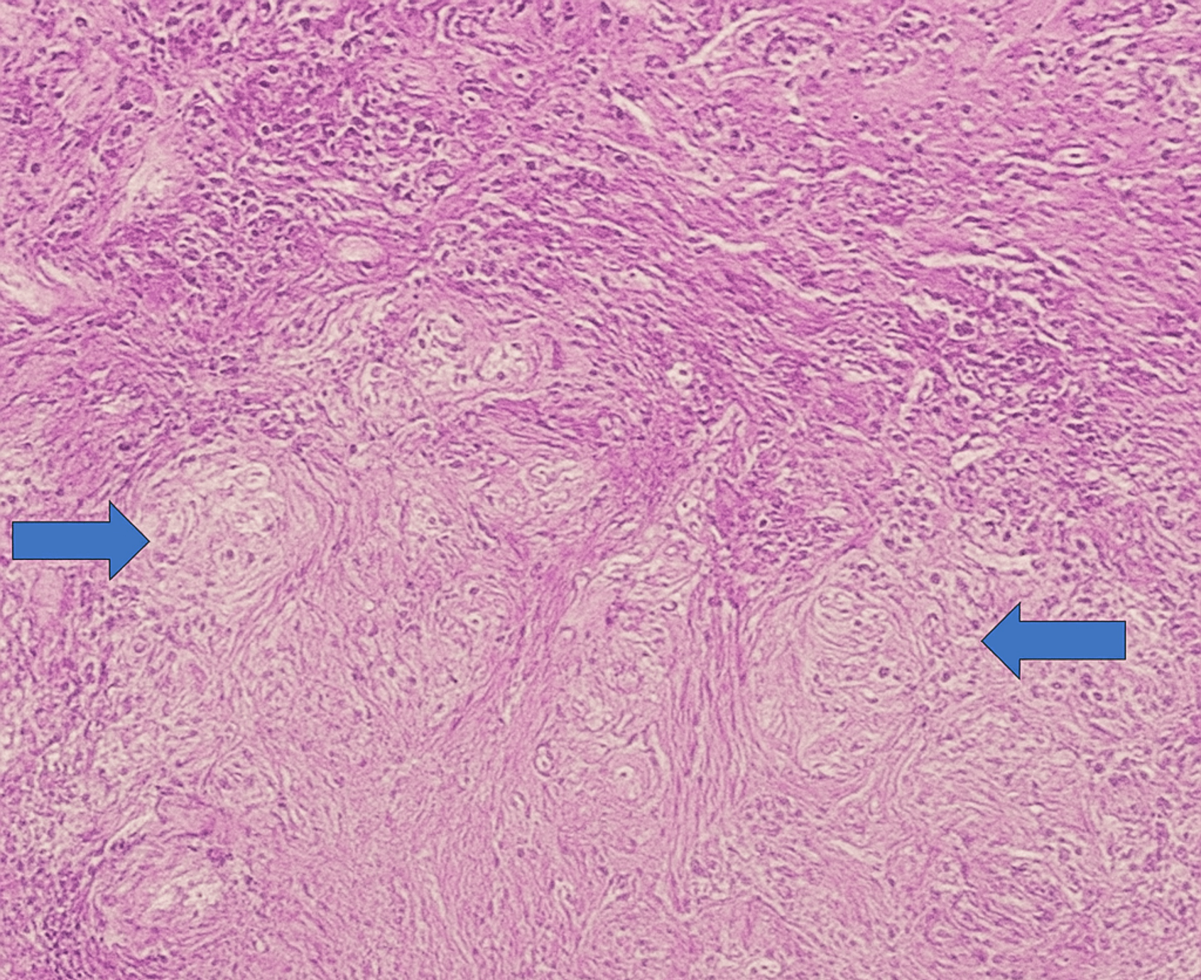
H&E stain, x200 Histological image showing multiple well-formed epithelioid granulomas (blue arrows) surrounded by lymphocytic infiltration, consistent with laryngeal tuberculosis. No evidence of cellular atypia is observed, helping to differentiate from malignant lesions.

The patient underwent direct laryngoscopy and biopsy under general anesthesia. Histopathological examination of the laryngeal lesion revealed caseating granulomas composed of epithelioid histiocytes and Langhans-type giant cells with central necrosis. Ziehl-Neelsen staining demonstrated acid-fast bacilli. Sputum PCR and culture confirmed infection with *Mycobacterium tuberculosis,* establishing a final diagnosis of LTB with pulmonary involvement (Figures 7-8).

Treatment was initiated with a standard four-drug anti-TB regimen consisting of isoniazid, rifampin, pyrazinamide, and ethambutol. The patient showed notable clinical improvement within a few weeks, including reduced dyspnea and hoarseness, along with weight stabilization. A follow-up laryngoscopic examination at two months revealed marked regression of the laryngeal lesions. The patient remained adherent to therapy and continued to progress favorably.

## Discussion

LTB is caused by *M. tuberculosis* and represents the most common granulomatous disease of the larynx. However, primary laryngeal involvement is rare, as the disease is almost always associated with active pulmonary TB [5-7].

Due to its non-specific laryngoscopic features, LTB can closely mimic laryngeal carcinoma. It often occurs following reactivation of a latent laryngeal focus, originally seeded during hematogenous dissemination of a primary infection. The condition typically affects adults in their 40s and 50s and presents with progressive hoarseness and odynophagia. Risk factors include chronic smoking, immunosuppression, HIV infection, malnutrition, poor socioeconomic conditions, and lack of BCG vaccination [5-7].

Laryngoscopy may reveal either ulcerative or mass-like lesions. The location is variable, but the true vocal cords are most commonly affected, followed by the posterior commissure, ventricular bands, epiglottis, and subglottic area. Lesions are bilateral in approximately 75% of cases [8].

Radiologic findings depend on the stage and extent of disease and correlate with histopathology. The infiltrative stage shows focal thickening, while the ulcerative stage presents with shallow mucosal ulceration that rarely involves cartilage or paraglottic spaces. Paralaryngeal fat planes are usually preserved, calcifications are rare, and perichondritis may occasionally occur, especially in the epiglottis or arytenoids. In advanced stages, sclerosis may develop.

Various radiological appearances have been described, including isolated edema, ulcero-infiltrative masses, pseudo-tumoral lesions (seen in ~66% of cases), subglottic swelling, cartilaginous ulceration, chondritis, or tuberculoma (a bulky ventricular mass elevating the ventricular band) [8-10].

Laryngeal carcinoma is the primary differential diagnosis. Unfortunately, imaging alone is often insufficient to distinguish between the two entities, necessitating histopathologic confirmation via biopsy. Table 1 summarizes the distinguishing features between LTB and laryngeal carcinoma.

**Table 1:** Difference between laryngeal tuberculosis from laryngeal carcinoma Sources: Refs [11,12]

Feature	Laryngeal Tuberculosis (TB)	Laryngeal Carcinoma
Age Group	More common in younger individuals or immunocompromised individuals	More common in older individuals, especially smokers and alcohol users
Symptoms	Hoarseness, painful swallowing, weight loss, fever, night sweats	Persistent hoarseness, pain, difficulty swallowing, weight loss
Laryngoscopic Findings	Ulcers, granulomas, intact cartilage	Irregular mass, ulceration, cartilage destruction
Radiological Findings	Thickened vocal cords, airway narrowing, preserved cartilage	Irregular lesions with possible cartilage destruction
Histopathology	Caseating granulomas, acid-fast bacilli	Malignant squamous cells, no granulomas
Treatment	Anti-tuberculous therapy (Rifampin, Isoniazid, Pyrazinamide, Ethambutol)	Surgery, radiotherapy, chemotherapy
Prognosis	Excellent with proper treatment	Depends on stage; poorer prognosis with advanced stages

Although imaging features raised suspicion of laryngeal carcinoma, further diagnostic tools such as PET-CT or laryngeal ultrasound were not deemed necessary. The lesion was readily accessible via laryngoscopy, allowing prompt biopsy and histological confirmation. Bronchoscopy was not performed, as pulmonary involvement was already confirmed through sputum PCR and culture.

Treatment is based on standard anti-TB therapy, and prognosis is generally favorable. However, surgical intervention may be required in cases of laryngeal stenosis [8].

## Conclusions

LTB should be considered in the differential diagnosis of patients presenting with laryngeal symptoms, particularly in regions with high TB prevalence or in individuals with relevant risk factors. Due to its ability to closely mimic laryngeal carcinoma in both clinical and radiological presentations, a high index of suspicion is essential. In our case, histopathological examination revealed caseating granulomas, confirming the diagnosis, and the patient showed significant clinical improvement after two months of anti-TB therapy. These findings underscore the importance of timely histopathological confirmation to avoid misdiagnosis and ensure appropriate management.
